# Gastric Biofeedback in Virtual Reality: Feasibility, Efficacy and Self-Reported Experience

**DOI:** 10.1007/s10484-025-09741-x

**Published:** 2025-10-23

**Authors:** Aline Tiemann, Zoé van Dyck, Claus Vögele, Simone Munsch, Marius Rubo

**Affiliations:** 1 Department of Psychology, Clinical Psychology and Psychotherapy, University of Fribourg, Av. de l’Europe 20, 1700 Fribourg, Switzerland; 2 Food Research and Innovation Center, FRIC, Cluster Food and Mental Health / Psychology, University of Fribourg, Av. de l’Europe 20, 1700 Fribourg, Switzerland; 3 Department of Behavioural and Cognitive Sciences, Institute for Health and Behaviour, University of Luxembourg, 2, place de l’Université, 4365 Esch-sur-Alzette, Luxembourg; 4 Department of Cognitive Psychology, Perception and Methodology, Institute of Psychology, University of Bern, Fabrikstrasse 8, 3012 Bern, Switzerland; 5 Centre Hospitalier Neuro-Psychiatrique, 17 Av. des Alliés, 9012 Ettelbruck, Luxembourg

**Keywords:** Gastric biofeedback, Virtual reality, Electrogastrography, Biofeedback

## Abstract

Normal gastric functioning (normogastria) is characterized by rhythmic gastric myoelectric activity, while dysrhythmic gastric activity (bradygastria and tachygastria) is associated with symptoms such as nausea, epigastric discomfort and fullness. Gastric biofeedback using electrogastrography (EGG) has emerged as a promising tool to regulate gastric rhythm, but research remains limited with few experimental studies. The current study (*N* = 94) tested a novel gastric biofeedback training in virtual reality (VR). We used a randomized controlled study design with three groups: (1) VR gastric biofeedback training, (2) the same training on a 2D screen and (3) a relaxation control group. We examined (1) the trajectory of gastric activity before, during, and after training (2) participants’ ability to increase normogastria and reduce dysrhythmic activity and (3) self-reported experiences (motivation, attention, mood, presence, user acceptance, etc.). Results showed the expected peak in normogastria and a nadir in dysrhythmic activity during training, with a significant reduction in dysrhythmic gastric activity across sessions. Participants reported an improved ability to handle physical discomfort over time. VR and 2D groups reported faster time perception and better concentration than controls. Spatial presence (i.e., the feeling of “being there”) was higher in the VR group than in the 2D group. Enjoyment, concentration, and alertness were initially high but declined over time in all groups. The results demonstrate the general technical feasibility of the paradigm and positive evaluations. Further research is needed to better understand the underlying mechanisms and to determine whether and how training success may relate to beneficial outcomes in clinical populations.

**Supplementary Information:**

The online version contains supplementary material available at 10.1007/s10484-025-09741-x.

## Introduction

### Gastric Biofeedback

The fundamental role of the stomach is digestion, by mechanically processing food through rhythmic contractions of its muscular walls. These movements are regulated by slow electrical waves that originate from a class of pacemaker cells, the interstitial cells of Cajal (ICCs; Koch & Stern, 2004). ICCs are functionally linked to the stomach’s smooth muscles, enabling the peristaltic activity necessary for gastric transit (Hunt et al., 2015; Janssen et al., 2011; Soybel, 2005). This myoelectrical activity can be captured non-invasively using electrogastrography (EGG), which records signals from electrodes placed on the abdominal surface (Koch & Stern, 2004; Wolpert et al., 2020). Simultaneous recordings using non-invasive surface EGG and invasive techniques in humans have demonstrated that EGG captures gastric myoelectrical activity in a manner comparable to direct measures (Hamilton et al., 1986; Lin et al., 2000; Mintchev et al., 1993). Although EGG signals are a combination of both the electrical oscillations from ICCs and the mechanical contractions of the stomach, the dominant frequencies are believed to largely reflect pacemaker activity (Wolpert et al., 2020).

A rhythmic gastric myoelectrical activity of 3 cpm (normogastria) is associated with normal gastric functioning. This 3 cpm rhythm underlies the coordination of gastric motility, enabling efficient digestion and gastric emptying. In healthy individuals, water or food intake typically leads to an increase in normogastria (Diamanti et al., 2003; van Dyck et al., 2020) and a decrease in dysrhythmic gastric activity (Diamanti et al., 2003; Mathur et al., 2001). It has been shown that relaxation increases normogastria (Yin et al., 2004), while physiological stress seems to increase dysrhythmic gastric activity (Muth et al., 1999; Yin et al., 2004).

Abnormal gastric functioning - typically presenting with symptoms such as nausea, epigastric discomfort and fullness - is linked to dysrhythmic gastric activity, namely bradygastria (1–2 cpm) or tachygastria (4–10 cpm) (Koch & Stern, 2004; Ogawa et al., 2004; van Dyck et al., 2020; Wolpert et al., 2020). Dysrhythmic gastric activity and accompanying symptoms such as nausea or abdominal discomfort has been observed in a number of conditions, ranging from eating disorders, depression, anxiety, schizophrenia, diabetes, functional dyspepsia, and vomiting syndromes (Carson et al., 2022; Diamanti et al., 2003; Janssen et al., 2011; Ly et al., 2015; Mathur et al., 2001; Ogawa et al., 2004; Peupelmann et al., 2009; Ruhland et al., 2008; Simpson & Stakes, 1987; van Dyck et al., 2020). Additionally, gastric motility and muscle tone influence perceptions of hunger, satiation, and fullness by mediating stomach distention (Janssen et al., 2011). Experimental increases in gastric muscle tone, which reduce the stomach’s capacity to relax, have been shown to elicit earlier satiation in healthy individuals (Janssen et al., 2011; Tack et al., 2002). These findings indicate that regulating the gastric rhythm, by increasing normal activity and reducing dysrhythmic patterns, may offer a promising approach to alleviate associated symptoms such as nausea, epigastric discomfort, and stress.

One possible method for this modulation may be gastric biofeedback (Stern et al., 2004; Vujic et al., 2020). During biofeedback training, individuals learn to voluntarily modify their physiological activity (more commonly heart functioning or breathing) as they receive continuous visual or auditory feedback on their physiological activity (Schwartz, 2010). To date, research on gastric biofeedback is scarce, although some study results are promising in terms of feasibility (Stern et al., 2004; Whitehead & Drescher, 1980). The only non-invasive approach with cutaneous electrodes (without the need for swallowing tubes or inflating gastric balloons, such as in Whitehead & Drescher, 1980) was conducted by Stern et al. (2004) and has not been replicated since. In this study, healthy young students (*N* = 26) were able to increase their normal 3 cpm gastric myoelectrical activity using gastric biofeedback of EGG signals (Stern et al., 2004). This lack of replication highlights the need to test whether biofeedback using gastric myoelectrical activity is feasible and effective.

### Virtual Reality Biofeedback

An additional question is how the mode of visualization may affect training effects in biofeedback training. A particularly immersive and attention-capturing form of visual representation can be achieved using virtual reality (VR; Cipresso et al., 2018; Freeman et al., 2017; Gradl et al., 2018). For multiple forms of biofeedback, VR implementations are gaining popularity. This includes, for example, biofeedback of heart rate variability (HRV), heart rate (HR), paced breathing or electrodermal activity (Lüddecke & Felnhofer, 2022). A recent comprehensive review reports higher motivation, involvement, attentional focus and more positive user experience in VR biofeedback compared to 2D feedback (Lüddecke & Felnhofer, 2022). We would assume, therefore, that self-reported evaluations are more positive in biofeedback involving VR than in those using other modes of implementation. These advantages may have a beneficial effect on training outcomes and may lead to lower dropout rates and higher compliance. Although some studies do indeed report better training outcomes for VR biofeedback (Rockstroh et al., 2019; Weibel et al., 2023) other researchers are more hesitant regarding this conclusion (Cortez-Vázquez et al., 2024; Lüddecke & Felnhofer, 2022; Pratviel et al., 2024).

One reason for this caution is the fact that the scene presented in the frequently used 2D control condition differed substantially from the VR condition. For instance, it often consisted of a simplistic feedback signal (e.g., a pulsating dot on a dark background; Rockstroh et al., 2019) or simply involved an inactive control group (e.g., waitlist condition; Kerr et al., 2023). These mismatches complicate the interpretation of the results, as differences between conditions could stem from discrepancies in sensory input or engagement effects, rather than from the immersive quality of the VR environment per se. Thus, more studies comparing the same visual feedback, such as a recent study by Weibel et al. (2023) and Pratviel et al. (2024) on different mediums, are necessary. To the best of our knowledge, there are no studies to date implementing a gastric biofeedback paradigm in VR.

## The Present Study

The goal of our study was to replicate the findings of Stern et al. (2004) regarding the feasibility and efficacy of gastric biofeedback in a group of healthy young adults, and to investigate potential additional effects of a novel form of visual representation using VR. In contrast to the study by Stern et al. (2004) where the EGG signal was visualized as a 2D wave signal, we implemented visual feedback into a nature environment. Virtual nature environments are a common design choice in the VR biofeedback field (Blum et al., 2019; Lüddecke & Felnhofer, 2022; Rockstroh et al., 2019; Weibel et al., 2023). Nature environments have been suggested to provide a favorable balance between promoting relaxation while being sufficiently engaging (Gaume et al., 2016; Rockstroh et al., 2019; Yoon & Jeon, 2025). As proposed by Rockstroh and colleagues (2019), we also considered attention restoration theory for nature environments. This theory emphasizes how certain environmental qualities can support cognitive recovery, such as the feeling of being away, stimuli that elicit involuntary attention or fascination, extension of space and to be aligned with one’s goals (here biofeedback; R. Kaplan & Kaplan, 1989; Kaplan, 1995). Therefore, our study design involved three experimental groups: (1) a VR gastric biofeedback group, (2) a 2D gastric biofeedback group (same scene as in VR, but displayed on a TV screen) and (3) a relaxation control group (CG). Biofeedback paradigms in VR are rarely compared with the same scene in 2D, with a few recent exceptions (Weibel et al., 2023). Therefore, the goal of the present paper was to assess the technical feasibility and efficacy as well as self-reported evaluations of the gastric biofeedback paradigm in VR, and to test whether it has advantages over the same 2D visualization and a relaxation condition in young healthy adults. Outcomes included (1) changes in the percentages of normogastria, bradygastria and tachygastria at baseline, during, and after the training, (2) group differences (VR, 2D and CG) in the percentages of normogastria, bradygastria and tachygastria across the four training sessions and (3) self-reported evaluations (including motivation, attention, mood, presence, user acceptance, etc.) between groups (VR, 2D and CG) across the four training sessions.

1. *Hypothesis 1* We expected normogastria to be lowest after fasting, to increase during the training and to decrease again after a water load test until fullness (as this may cause nausea, and nausea is associated with less normogastria and more dysrhythmic activity). Respectively, we expected bradygastria and tachygastria to decrease from baseline to training, and to increase again following water ingestion until fullness.
2. *Hypothesis 2* We expected participants in the VR group to show more pronounced increases and reductions in normogastric and dysrhythmic activity, respectively, across sessions than participants in the 2D group. We also hypothesized that participants in the 2D group would respond with stronger increases and reductions in normogastric and dysrhythmic activity, respectively, compared to the CG.
3. *Hypothesis 3* We expected that participants in the VR group would show the most positive self-reported evaluations, followed by the 2D group and finally the CG.

## Methods

### Power Analysis and Participants

We used G*Power to conduct an a priori power analysis. To achieve a medium effect size (*f* = 0.15), with 3 groups (VR, 2D and CG) and 4 repeated measurements (4 sessions) a sample size of *N* = 78 is required (*α* = 0.05; 1–*β* = 0.80). The study procedure, sample size, hypothesis 2 and hypothesis 3 were pre-registered (10.17605/OSF.IO/JSKPZ). In addition, we also reported on the trajectory of gastric activity before, during, and after training (hypothesis 1). 96 participants were recruited at the University of Fribourg through university platforms and flyers. Two participants were excluded because their four training sessions were more than two weeks apart, resulting in 94 participants (73 females, 21 males). Due to technical issues, data of 9 participants could not be used for the Igroup Presence Questionnaire (Schubert et al., 2001). For being included in the study, participants needed to be at least 18 years of age. Exclusion criteria included a history of gastrointestinal illnesses or surgeries, medication that may modify gastric myoelectrical activity (e.g., prokinetics, antiemetics, narcotics, anticholinergic drugs, and nonsteroidal anti-inflammatory drugs), psychotropic medication, bipolar disorder, current or past psychotic disorders, current suicidal ideation, epilepsy, physical conditions or treatments affecting eating behavior or body weight. (e.g., diabetes mellitus) and pregnancy or breastfeeding. These exclusion criteria were based on similar studies using similar techniques/tasks (Blum et al., 2019; van Dyck et al., 2020) and recent guidelines for EGG by Wolpert et al. (2020). Ethical approval was obtained from the Internal Ethics Review Board of the Department of Psychology of the University of Fribourg (2023 − 824 R1) and the study was conducted in accordance with the Declaration of Helsinki. Participants provided written informed consent before the start of the study.

### Procedure

**Fig. 1:**
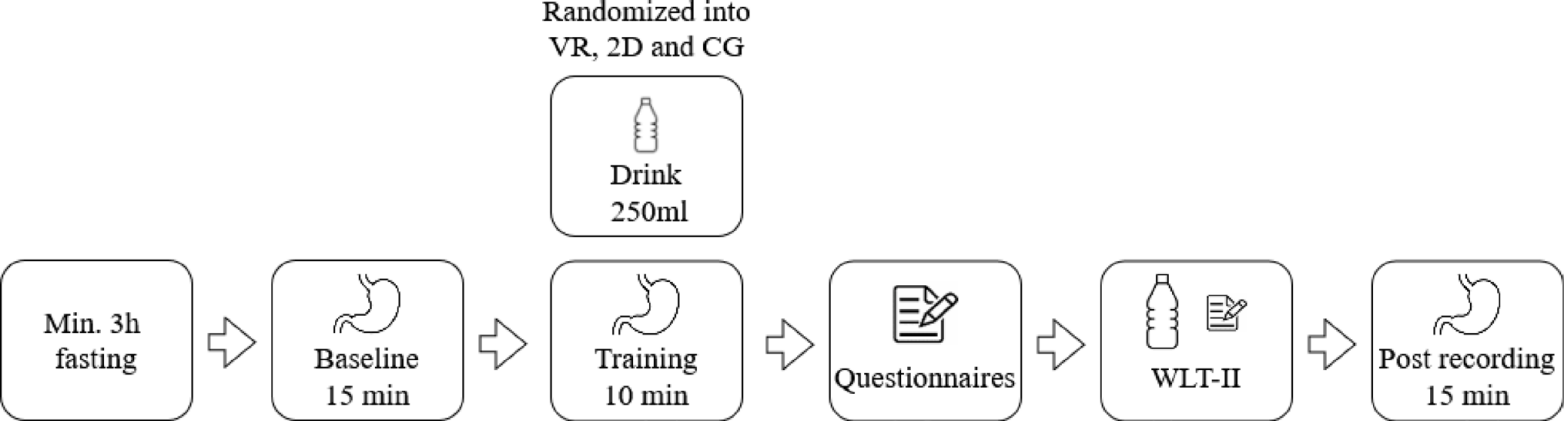
Study procedure

Participants were asked to fast and refrain from consuming any beverages for at least 3 h before the study. They were compensated with a snack after the study and with study participation credits if applicable. Participants were randomly allocated to one of the following groups via block randomization (1) VR gastric biofeedback paradigm (*n* = 30), (2) 2D gastric biofeedback paradigm (*n* = 32) or (3) a relaxation CG (*n* = 32). The study consisted of four laboratory sessions within 2 weeks. Upon arriving in the laboratory, participants underwent a 15 min baseline rest EGG measurement. Participants then drank 250 ml of water, followed by a 10 min training session (VR, 2D or CG). After the training, participants completed questionnaires. Finally, they performed the two-step water load test (van Dyck et al., 2016) involving water intake until fullness, followed by another 15 min rest EGG. The same procedure was repeated in sessions 2, 3 and 4. The study procedure is depicted in Fig. 1.

### Materials

**Fig. 2:**
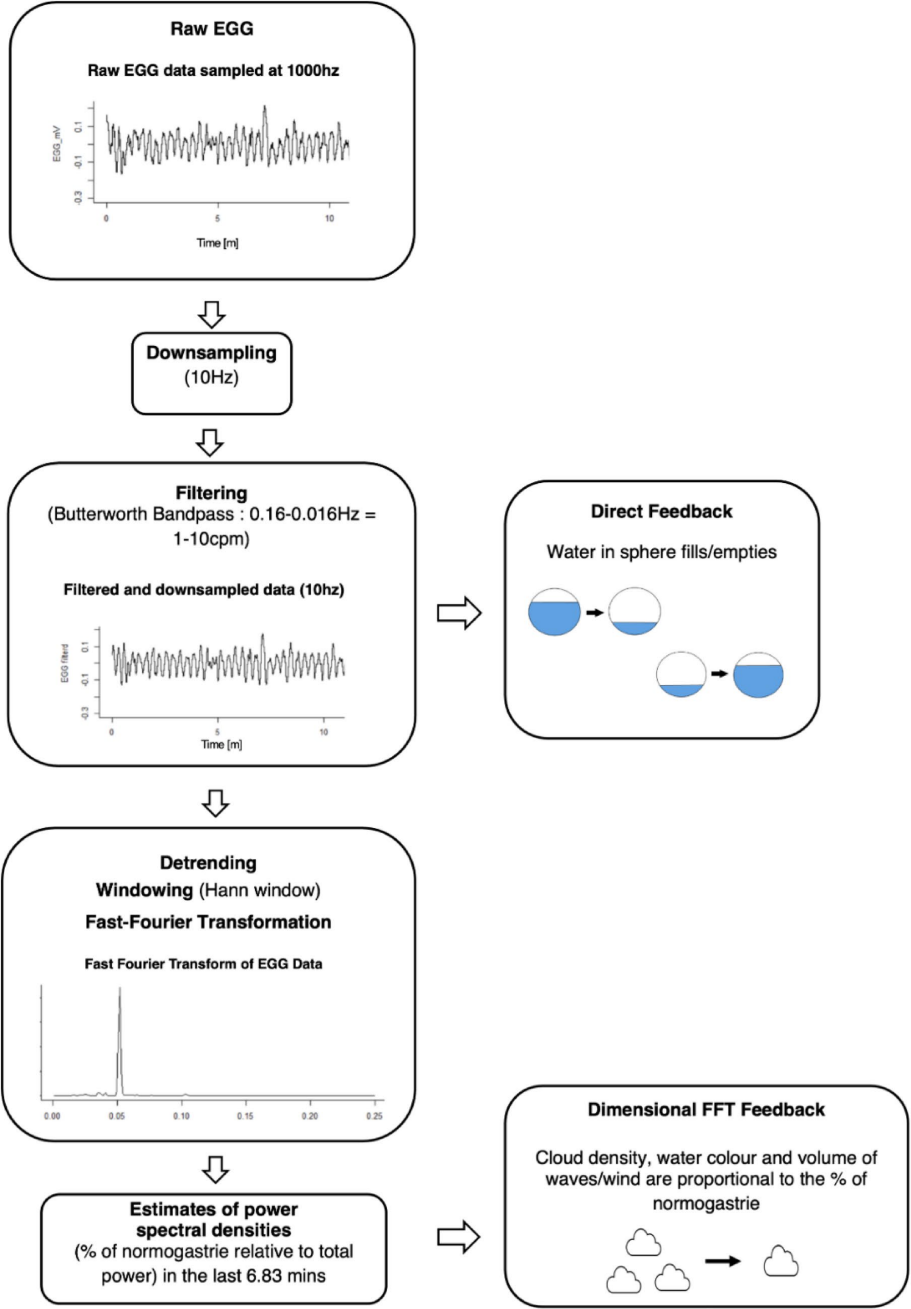
Signal processing for the direct feedback elements (transparent sphere) and the dimensional Fast Fourier Transform feedback elements (weather / environment). The raw EGG signal was sampled at 1000 Hz. The signal was then downsampled to 10 Hz and filtered with a bandpass Butterworth filter with a frequency range of 0.016–0.16 Hz. This signal was used for the direct feedback. The signal was then detrended, Hann-windowed and Fast Fourier transformed. The plot shows the frequency components of normogastric activity with a peak frequency around 0.05 Hz. Finally, the result of the power spectral density estimatiom was used for the dimensional Fast Fourier transformed feedback

In our experimental setup, we employed a Windows 10 computer with 32 gigabytes of RAM, an Intel Core i5-12600 K processor and a Nvidia GeForce RTX 3060 Ti graphics card. For the VR condition, participants watched the gastric biofeedback scene through an HTC Vive XR Elite VR headset (www.vive.com), with a resolution of 1920*1920 pixels per eye and a frame rate of 90 frames per second. In the 2D condition, participants were seated in front of a 31.5-inch TV screen, at a distance of 120 centimeters. During the baseline EGG measurement and during the VR, 2D or CG training session, all participants sat in a Lafuma RSX reclining chair (www.lafuma.com). Gastric myoelectric activity was measured using a Bluetooth BITalino device (Batista et al., 2019; da Silva et al., 2014) with a sampling rate set to 1000 Hz and a 16-bit resolution. The gastric biofeedback paradigm was programmed using the Unity3D game engine (https://unity.com/). EGG raw data were livestreamed to the VR application using the PLUX Unity API (https://github.com/pluxbiosignals/unity-sample), and custom C# scripts were written to process real-time EGG data and translate them into the biofeedback visualization for participants (see Fig. 2). The same data processing was used for the baseline, training and post-training EGG recordings.

### EGG Baseline and Post Training Recordings

**Fig. 3:**
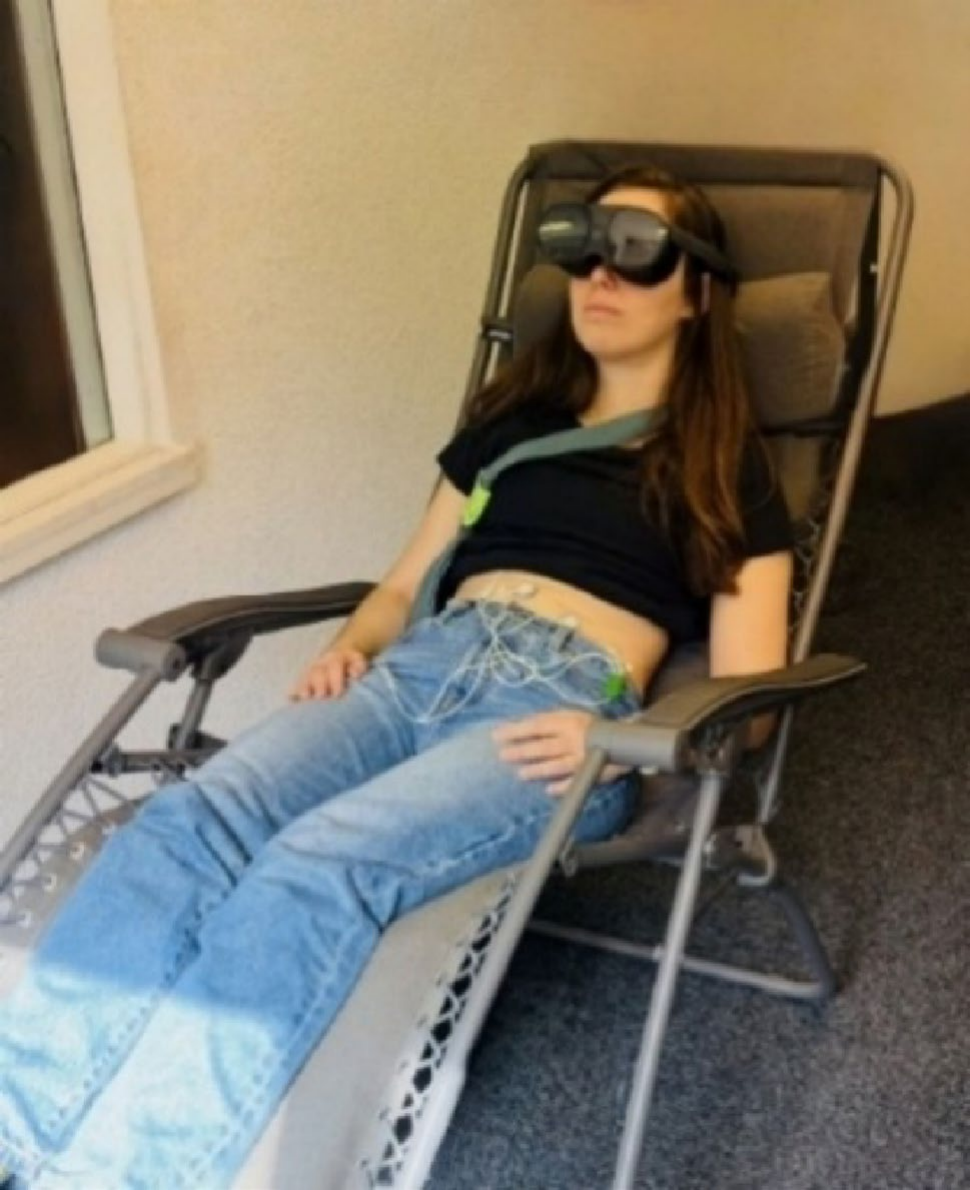
Experimental setup for the VR gastric biofeedback paradigm. The participant is seated in a reclining chair, wearing a VR headset and equipped with EGG electrodes

For the 15 min rest EGG periods (baseline and after the training), we used the OpenSignals (r)evolution software for signal acquisition, a recommended choice for BITalino. Prior to electrode placement, respective areas of the skin were cleaned with alcohol and gently abraded using Nuprep gel (D.O. Weaver and Co., Aurora, CO, USA). As detailed in guidelines by Riezzo et al. (2013), we placed three abdominal electrodes (ConMed Cleartrace) over the gastric antrum region. The first active electrode was carefully positioned midway between the umbilicus and the xiphoid notch, while the second one was applied approximately 5 cm to the right at an upper 45 degree angle. The ground electrode was placed on the left costal margin. During EGG recording, participants assumed a semi-reclining position in a comfortable chair tilted at an angle of approximately 30–45° (see Fig. 3). Participants were instructed to remain silent and minimize body movements throughout the recording process.

Data preprocessing of the two 15 min rest EGG periods was conducted using R (version 4.3.2, R Core Team, 2024), incorporating established data processing procedures (Koch & Stern, 2004; van Dyck et al., 2020; Wolpert et al., 2020). Signal data were visually inspected to assess overall quality and identify potential artifacts. Only continuous recordings devoid of artifacts and characterized by visually discernible waveforms were retained for analysis. The data was then down-sampled to a rate of 10 Hz, subjected to detrending, and filtered using a bandpass Butterworth filter with a frequency range of 0.016 to 0.16 Hz (equivalent to 1–10 cpm as per Koch & Stern, 2004). We then applied a Hanning window and zero-padding to the nearest power of 2. To extract distinct frequency power bands, we performed a Fast Fourier Transformation (FFT). Specifically, we extracted the following power bands: bradygastria (1–2 cpm), normogastria (2–4 cpm), and tachygastria (4–10.0 cpm) (as proposed by Wolpert et al., 2020). The power within each EGG band was subsequently computed as a percentage proportion of the total EGG band power within its corresponding frequency range.

### Two Step Water-Load Test (WLT-II; Van Dyck et al., 2016)

Post-training *nausea* (*before* water ingestion) was measured via an item on the two-step water load test questionnaire (van Dyck et al., 2016). Response options consisted of a 7-point Likert scale (1 = *no nausea*, 7 = *nausea*). The WLT-II was administered after the training sessions. We used the WLT-II to measure changes in EGG frequency bands between the fasted state, training state and fullness state (after WLT-II, post-training, which might induce nausea and therefore increase dysrhythmic activity). The WLT-II consists of drinking water in two steps. First, participants drink water until satiation and then, in a second step, continue to drink until they experience fullness. The procedure is described in detail in (van Dyck et al., 2016).

### Gastric Biofeedback Paradigm (VR and 2D)

**Fig. 4:**
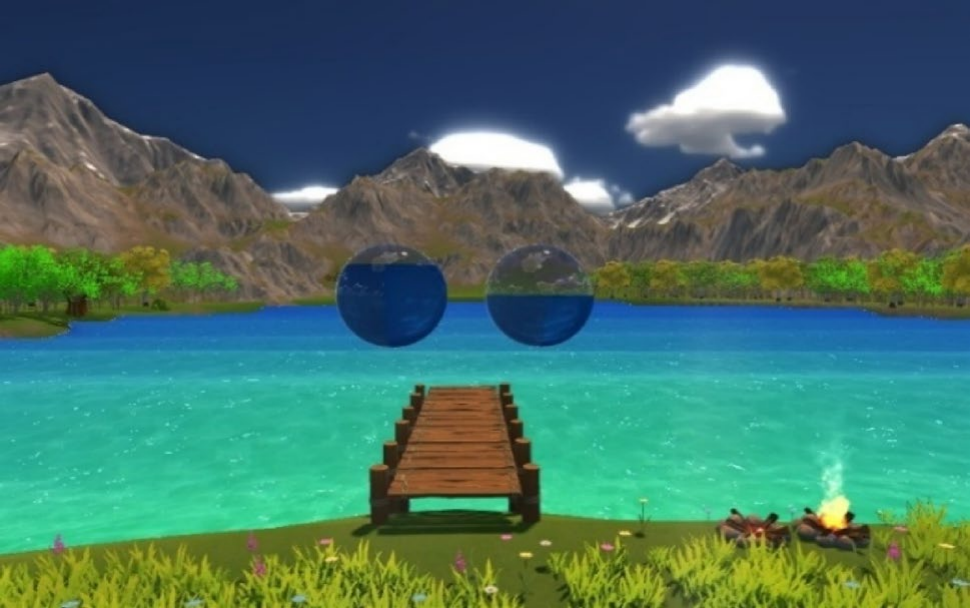
Virtual nature environment depicted in the gastric biofeedback paradigm

Participants viewed biofeedback embedded into a virtual nature environment. For both the VR and 2D groups, the experimental setup remained the same, differing only in the display medium (VR headset vs. television). Participants viewed an immersive nature environment featuring a lake and mountains (see Fig. 4), designed to provide biofeedback through different visual elements. Participants received direct feedback (filtered raw signal) of their stomach activity, where two transparent spheres filled and drained with water in a rhythmic manner, floating above the lake. The left sphere acted as a pacemaker, maintaining a steady rhythm of 3 cpm (corresponding to the normogastric rhythm), while the right sphere reflected the participant’s own EGG activity in real time (see Fig. 2). Participants also received dimensional FFT Feedback, in which participants’ percentage of normogastric activity was represented through environmental changes, such as cloud density, water color, and variations in wind and water sounds (see Fig. 2). Higher normogastria resulted in sunnier weather with clearer skies.

Participants were instructed in German (see Supplementary material S1), and were guided to use relaxation and controlled breathing to influence their own stomach activity (sphere on the right), aligning it with the pacemaker sphere’s rhythm (sphere on the left). They were encouraged to focus on calm breathing and avoid stressing over perfect synchronization, as progress was indicated by the right spheres’ movement and increasingly pleasant weather conditions.

### Relaxation Control Group (CG)

The EGG data of participants in the CG was recorded in Unity and processed through the same pipeline as the other two groups. However, they did not see the scene and instead received spoken instructions (see Supplementary material S1), to use relaxation and mental effort to influence their stomach activity, aiming to match the normal rhythm of three contractions per minute. They were guided to achieve this by breathing calmly, thinking of pleasant imagery like a beach day, and visualizing their stomach contracting like a balloon or water-filled ball inflating and deflating three times per minute, while minimizing movement and abdominal muscle contractions.

### Questionnaires

#### Satisfaction with the Gastric Biofeedback Paradigm

Three aspects of the gastric biofeedback paradigm were assessed on a visual analogue scale (VAS) from 1 to 10. This included a question about relaxation “*I found the scene relaxing*”, one on the visualization of the direct feedback “*I found the ball to be an intuitive visualization of my stomach activity*” and a final one on the FFT feedback “*I found the environmental visualizations (clouds*,* water*,* sounds) helpful in understanding my stomach activity*”. These questions were only administered in the two biofeedback (VR and 2D) groups, not in the CG.

#### General State Questions for Biofeedback Based on Rockstroh et al. (2019)

Motivational aspects and attentional focus were assessed based on items proposed by Rockstroh et al. (2019). Motivational aspects were measured with four questions regarding *general liking* (0 = *not at all*, 10 = *a lot*), *intention to use* (0 = *rarely*, 10 = *very often*), *recommendation to others* (0 = *not at all*, 10 = *very strongly*) and *time perception* (0 = *very slowly*, 10 = *very quickly*). Attentional focus was assessed with two items, *concentration* and *distraction* (0 = *not at all*, 10 = *completely*).

#### Multidimensional Mood Questionnaire (MDBF; Steyer et al., 1997)

Participants’ mood was assessed with the MDBF. The MDBF consists of two parallel versions, making it useful for repeated measurements. Versions A and B were randomized. The questionnaire has three subscales, each consisting of four items, on a five-point Likert scale (1 = *not at all*, 5 = *very much*): *calmness versus restlessness (RU)*, *good versus bad mood (GS)* and *alertness versus fatigue (WM)*. Higher values indicate better mood.

#### Igroup Presence Questionnaire (IPQ ; Schubert et al., 2001)

The sense of presence that individuals experience in a virtual environment was assessed with the IPQ. It consists of 14 items and three subscales (Likert Scale 1–6, with higher values indicating higher agreement): *spatial presence (SP;* 5 items*)*, which refers to feelings of physical presence in the virtual environment, *involvement (INV;* 4 items*)* measuring both attention to and involvement with the virtual scene, *experienced realism (REAL;* 4 items) to capture how real the environment is experienced and lastly, a single item measuring the sense of being there. The IPQ was also only administered in the VR and 2D groups to avoid confusion in the CG group.

#### Virtual Reality Sickness Questionnaire (VRSQ; Kim et al., 2018)

Motion sickness symptoms in VR environments was assessed through the VRSQ. The questionnaire has two subscales: *oculomotor* (4 items: general discomfort, fatigue, eyestrain, difficulty focusing) and *disorientation* (5 items: headache, fullness of head, blurred vision, dizzy (eyes closed), vertigo). Answering options are on a 4-point Likert scale (1 = *not at all*, 4 = *very*).

#### User Acceptance Questionnaire for Biofeedback (UAQ; Klewinghaus & Martin, 2022)

User acceptance of the gastric biofeedback paradigm was assessed with the UAQ, which was designed to measure user acceptance of HRV biofeedback training. The questionnaire consists of seven items on a five-point Likert-scale (1 = *not at all*, 5 = *very strongly*). The questionnaire captures different aspects of user acceptance, including the influence of the biofeedback training on mood, stress, bodily state and bodily symptoms. It also assesses how satisfied participant are with the biofeedback training, how much they would transfer their acquired skills to everyday life and whether they experienced any unpleasant side effects.

### Data Analysis

Statistical analyses were carried out with IBM SPSS Statistics Version 26 (SPSS Inc. Chicago, IL) and R version 4.3.2 (R Core Team, 2022) for Windows 11. The R packages *lme4* (Bates et al., 2015), *lmerTest* (Kuznetsova et al., 2017), *emmeans* (Lenth, 2025), and *ggplot2* (Wickham, 2016) were used for data analysis and visualization. For EGG data, outliers above or below three standard deviations from the group mean were set to their respective upper or lower value of three standard deviations.

For *hypothesis 1* (differences in EGG activity before, during and after training between groups), mixed-effects models were fitted to examine the fixed effects of group (VR, 2D and CG), and timepoint (pre-training, during training, post-training) for normogastria, bradygastria and tachygastria, and their interaction, with random intercepts for participants to account for repeated measures.

For *hypothesis 2* (changes of EGG activity during training along the four sessions between groups), we fitted mixed-effects models to examine the fixed effects of group (VR, 2D and CG) and session (sessions 1,2,3 and 4) for normogastric, bradygastric and tachygastric activity, and their interaction, with random intercepts for participants to account for repeated measures.

For *hypothesis 3* (differences in self-reported experiences between groups), we fitted mixed-effects models to examine the fixed effects of group (VR, 2D and CG) and session (sessions 1,2,3 and 4) and their interaction for self-report variables, again with random intercepts for participants to account for repeated measures.

Significance was set to 0.05. To follow up on significant effects, we used post-hoc pairwise comparisons using the estimated marginal means (EMM) with adjusted p-values through Tukey’s HSD, in addition to effect sizes calculated with Cohen’s d using the *effsize* package (Torchiano, 2020). Prior to the analyses, dependent variables were z standardized. Socio-demographic characteristics of participants can be found in Table 1.

## Results

### Demographic characteristics

**Table 1:** Socio-demographic characteristics of participants

	VR (*n* = 30)	2D (*n* = 32)	CG (*n* = 32)	Total (*n* = 94)
Sex				
Female	26 (86.7%)	21 (65.6%)	26 (81.3%)	73 (77.7%)
Age M (SD)	22.83 (3.53)	26.13 (12.38)	24.34 (7.05)	24.47 (8.57)
BMI M (SD)	21.78 (2.37)	22.74 (3.01)	22.75 (2.79)	22.44 (2.75)

2D, 2D Group (television); BMI, Body Mass Index; VR , Virtual Reality Group; CG, Control Group; M, Mean; SD, Standard Deviation.

### Descriptives

Descriptive statistics of the variables included in the analyses can be found in Table 2 in the Supplement S2.

### Hypothesis 1: EGG Timepoint and Group Analysis

#### Normogastria

**Fig. 5:**
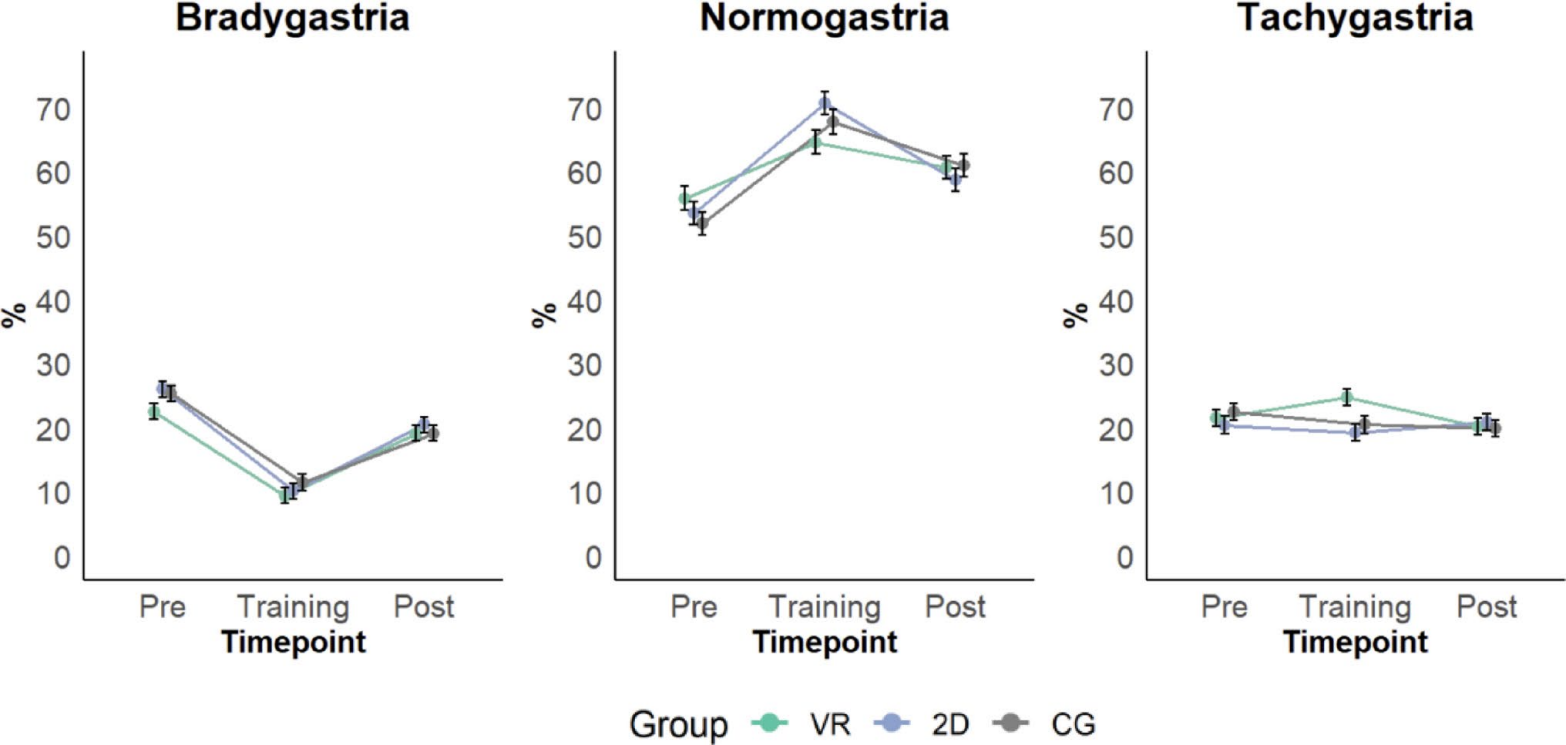
Bradygastria, Normogastria and Tachygastria for the VR, 2D and CG groups before, during and after the gastric biofeedback training. Values were aggregated across the 4 sessions. Error Bars represent standard error of the mean (SEM)

*Normogastric activity* significantly differed between Timepoints (*F*(2, 966.93) = 86.56, *p* < .001). Scores were significantly lower pre-training compared to during training (*p* < .001, *d* = −1.00, 95% CI [−1.15, −0.85]) and lower pre-training compared to post-training (*p* < .001, *d* = −0.46, 95% CI [−0.61, −0.31]). Conversely, scores were significantly higher during training compared to post-training (*p* < .001, *d* = 0.55, 95% CI [0.39, 0.69]) (see Fig. 5). Normogastria did not significantly differ between Groups (*F*(2, 93.81) = 0.07, *p* = .937), albeit the interaction between Group and Timepoint was significant (*F*(4, 966.70) = 4.25, *p* = .002) (see Table 3 in supplement S3).

#### Bradygastria

*Bradygastria* significantly differed between Timepoints (*F*(2, 968.93) = 184.00, *p* < .001). Scores were significantly higher pre-training compared to during training (*p* < .001, *d* = 1.44, 95% CI [1.29, 1.59]) and significantly higher pre-training compared to post-training (*p* < .001, *d* = 0.50, 95% CI [0.36, 0.66]). Conversely, scores were significantly lower during training compared to post-training (*p* < .001, *d* = −0.93, 95% CI [−1.09, −0.78]) (see Fig. 5). Neither did bradygastric activity differ between Groups (*F*(2, 94.29) = 1.11, *p* = .335) nor was the interaction between Group and Timepoint significant (*F*(4, 968.66) = 1.20, *p* = .310).

#### Tachygastria

*Tachygastria* did not significantly differ between Timepoints (*F*(2, 966.77) = 1.30, *p* = .272) or Groups (*F*(2, 91.68) = 0.86, *p* = .429). Nevertheless, the interaction between Timepoint and Group was significant (*F*(4, 966.50) = 3.33, *p* = .010), with significantly lower values of tachygastric activity during training compared to after for the VR group (*p* = .004, *d* = 0.41, 95% CI [0.16, 0.67]) (see Fig. 5). The difference between the VR group and 2D group during training was marginally significant (*p* = .09, *d* = 0.50, 95% CI [0.16, 0.83]), with the 2D group showing marginally significantly lower percentages of tachygastria than the VR group.

### Hypothesis 2: EGG Group and Session Effects

#### Normogastria

**Fig. 6:**
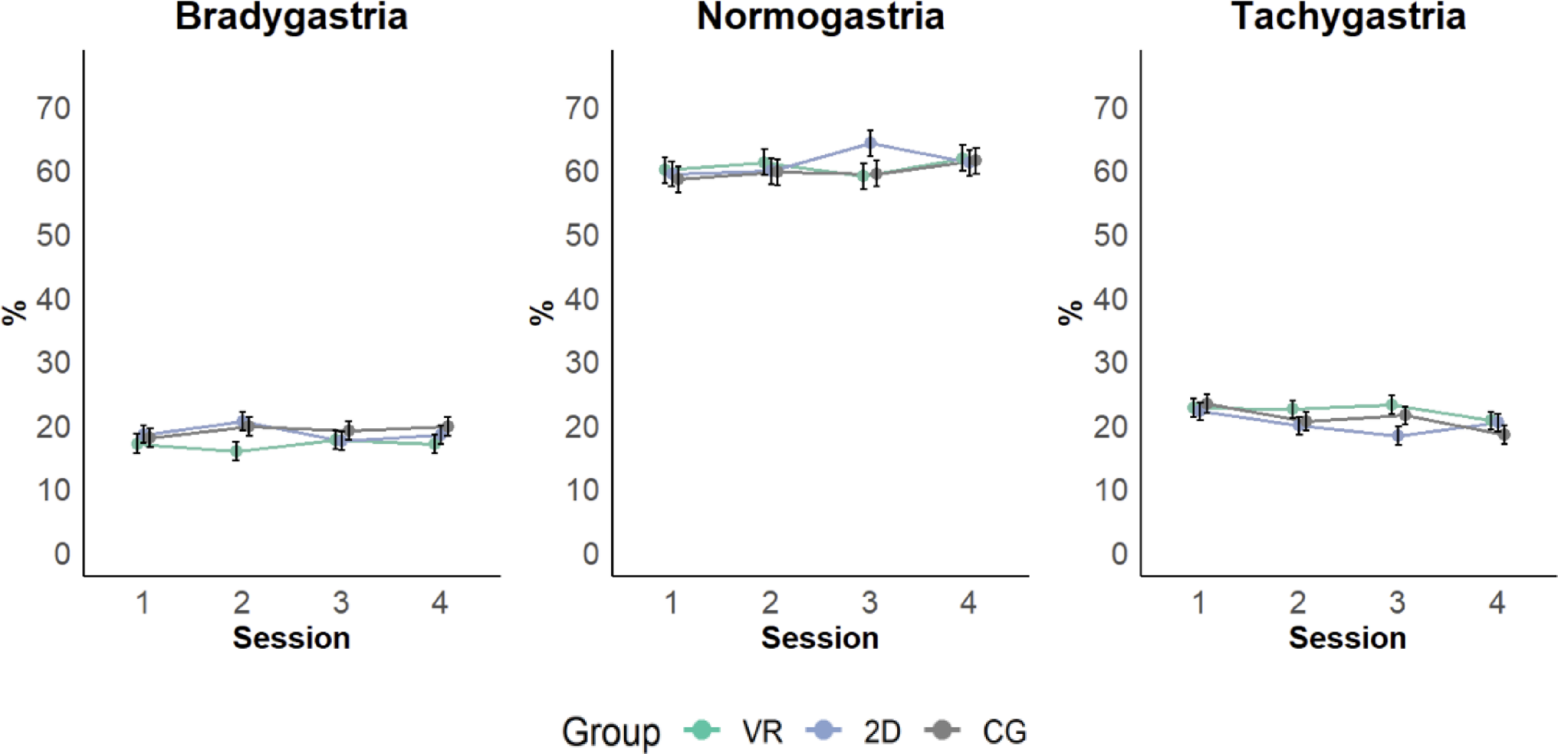
Bradygastria, Normogastria and Tachygastria for the VR, 2D and CG groups during the gastric biofeedback training. Error bars indicate standard error of the mean (SEM)

*Normogastria* did not differ between Sessions (*F*(3, 959.35) = 0.99, *p* = .394) or Groups (*F*(2, 92.51) = 0.22, *p* = .801), nor was there an interaction effect between Session and Group (*F*(6, 960.32) = 0.98, *p* = .435) (see Fig. 6).

#### Bradygastria

*Bradygastria* did not significantly between Sessions (*F*(3, 962.15) = 0.28, *p* = .838 ) and Groups (*F*(2, 93.22) = 1.55, *p* = .217). The interaction effect between Session and Group was not significant either (*F*(6, 963.54) = 0.77, *p* = .596) (see Fig. 6).

#### Tachygastria

*Tachygastria* significantly differed between Sessions (*F*(3, 958.14) = 3.20, *p* = .023). Scores in Session 1 were significantly higher compared to Session 4 (*p* = .011, *d* = 0.27, 95% CI [0.10, 0.44]) (see Fig. 6). Tachygastria did not differ between Groups (*F*(2, 91.26) = 0.99, *p* = .375) and there was no interaction effect between Session and Group (*F*(6, 959.13) = 1.33, *p* = .240).

### Hypothesis 3: Self-reported Experience Variables

The results of the mixed models are presented in detail in Table 4 in the supplement S4. Here, we report the post-hoc comparisons derived from these models.

#### Satisfaction with the Gastric Biofeedback Paradigm

There were no significant Timepoint, Group or Timepoint*Group effects for *how relaxing participants found the scene* and *how intuitive they found the visualization of their stomach activity* (see Table 4 in supplement S4). There was a significant main effect of Session for ratings of *perceived helpfulness of the environmental visualizations (clouds*,* water*,* sounds) in understanding stomach activity*. Scores were significantly lower in Session 1 compared to Session 3 (*p = .*009, *d =* −0.58, 95% CI [−0.94, −0.22]) and in Session 2 compared to Session 3 (*p = .*019, *d =* −0.53, 95% CI [−0.89, −0.17]). The difference between Session 1 and Session 4 was marginally significant, with scores being lower in Session 1 (*p = .*094, *d =* −0.42, 95% CI [−0.78, −0.06]). No significant effects were observed for Group or Timepoint × Group (all *p* > .05; see Table 4 in supplement S4).

#### Motivational Aspects

We found a significant main effect of Session for *general liking* of the program. Scores for *general liking* were significantly higher in Session 1 compared to Session 3 (*p* = .023, *d =* 0.42, 95% CI [0.13, 0.71]) and Session 4 (*p = .*021, *d =* 0.43, 95% CI [0.14, 0.71]). No significant differences were observed between the other sessions (all *p* > .05). There was no Group or Session × Group effect (see Table 4 in supplement S4). There were no Session, Group or Session × Group effects for *Intention to use* or *Recommendation to others* (see Table 4 in supplement S4). For *time perception*, the main effect of Session was marginally significant and there was a significant main effect of Group. Time was perceived as passing significantly faster in the 2D group compared to the CG (*p = .*042, *d =* 0.59, 95% CI [0.11, 1.07]) and the difference between the VR and CG was marginally significant, with time passing faster in the VR group (*p = .*084, *d =* 0.53, 95% CI [0.04, 1.02]). No significant differences were observed between the other Session or Group comparisons and the interaction between Session and Group was not significant (all *p* > .05; see Table 4 in supplement S4).

#### Attentional Focus

For *concentration*, we found significant main effects of Session and Group. Individuals indicated being significantly more concentrated in Session 1 compared to Session 3 (*p = .*018, *d =* 0.43, 95% CI [0.14, 0.72]) and Session 4 (*p* < .001, *d =* 0.63, 95% CI [0.34, 0.92]). They also indicated being significantly more concentrated in Session 2 compared to Session 4 (*p = .*008, *d =* 0.47, 95% CI [0.18, 0.76]). The difference in concentration between the VR and CG was marginally significant (*p = .*064, *d =* 0.62, 95% CI [0.08, 1.16]), and the 2D group compared to the CG was also marginally significant (*p = .*093, *d =* 0.57, 95% CI [0.03, 1.10]). No significant differences were observed between the other Session or Group comparisons and the Session by Group interaction was not significant (all *p* > .05; see Table 4 in supplement S4). For *distraction*, there were no significant Session, Group or Session ×Group effects (see Table 4 in supplement S4).

#### Mood: Multidimensional Mood Questionnaire (MDBF; Steyer et al., 1997)

For how *alert* individuals indicated to be, there was a significant effect of Session. Participants reported significantly more alertness in Session 1 compared to Session 3 (*p* = .007, *d =* 0.47, 95% CI [0.19, 0.76]) and Session 4 (*p* = .025, *d =* 0.42, 95% CI [0.13, 0.70]). The difference in alertness between Session 1 and Session 2 was marginally significant, with people being more alert in Session 1 (*p* = .056, *d =* 0.37, 95% CI [0.08, 0.66]). No significant differences were observed between the other Session comparisons and there was no significant main effect of Group or Session × Group (all *p* > .05). There was no significant main effect of Session, Group or Session × Group for *good mood* and *Rest (unrest)* (see Table 4 in supplement S4).

#### Presence: Igroup Presence Questionnaire (IPQ ; Schubert et al., 2001)

For *spatial presence*, there was a significant effect of Group, with individuals in the VR group reported higher levels of spatial presence than individuals in the 2D group (*p = .*005, *d =* 1.22, 95% CI [0.37, 2.07]). However, there was no significant effect of Session or Session × Group. There was no significant Session, Group or Session × Group effect of *involvement* or *realness* for the VR and 2D groups (see Table 4 in supplement S4).

#### VR Sickness Virtual Reality Sickness Questionnaire (VRSQ; Kim et al., 2018)

For the VRSQ, there was a marginally significant effect for Session, there was no significant main effect of Group, but there was a significant interaction between Session and Group, with significantly lower levels of VRSQ values in the CG in session 1 compared to session 2 (*p = .*020, *d =* 1.06, 95% CI [0.47, 1.64]) (see Table 4 in supplement S4).

#### Nausea

For nausea, there was a significant effect for Session. Participants reported significantly lower nausea in Session 4 compared to Session 1 (*p = .*001, *d =* 0.55, 95% CI [0.26, 0.83]). There was no significant effect for Group or Session × Group (see Table 4 in supplement S4).

#### User Acceptance Questionnaire for Biofeedback (UAQ; Klewinghaus & Martin, 2022)

Participants reported that the training *improved how they dealt with physical discomfort (UAQ1)*, with a significant main effect of Session. Ratings significantly increased from Session 1 to Session 3 (*p* = .052, *d* = −0.38, 95% CI [−0.67, −0.09]) and Session 4 (*p* < .001, *d* = −0.61, 95% CI [−0.90, −0.32]). Significant differences were also observed between Session 2 and Session 4 (*p* < .001, *d* = −0.61, 95% CI [−0.90, −0.32]), and was marginally significant compared to Session 3 (*p* = .058, *d* = −0.37, 95% CI [−0.66, −0.08]). No significant differences were found between Session 3 and Session 4 (*p* = .362, *d* = −0.24, 95% CI [−0.53, 0.05]). However, no significant main effect was found for Group or the interaction between Session and Group. For *UAQ 2–7*, there were no significant effects (see Table 4 in supplement S4).

## Discussion

The present study examined the general technical feasibility, efficacy and self-reported experience of a novel gastric biofeedback paradigm, comparing a VR group, a 2D group and a CG. In line with our hypotheses, normogastria significantly increased during training compared to baseline and significantly decreased again post-training, while bradygastria followed an inverse pattern. Contrary to our hypothesis, we found no significant differences in the trajectory of EGG measures between experimental conditions, but a trend for tachygastria, which was lower in the 2D group than in the VR group during the training (medium effect, marginally significant). Additionally, tachygastria significantly decreased across sessions. We found no group differences for normogastria and bradygastria during training. As hypothesized, regarding self-reported assessment measures, both VR and 2D groups perceived time as passing more quickly and reported higher concentration compared to the CG. Additionally, spatial presence was higher in the VR group than in the 2D group. Contrary to our hypothesis, we did not find the VR condition to be superior to the 2D group, and the 2D group to be superior to the CG for the remaining self-reported assessment measures. However, participants found the visualization of their stomach activity increasingly helpful over the course of the sessions and reported improved ability to manage physical discomfort across groups. Enjoyment, concentration, and alertness were initially high across groups but declined over time, possibly reflecting the absence of clinical need or treatment motivation among the healthy participants, which could differ in patient populations.

The first hypothesis could be confirmed for normogastria and bradygastria. Normogastria followed the expected trajectory, with scores peaking during training after a significant increase from pre-training and decreasing significantly post-training. The lower pre-training scores can be attributed to participants fasting at least three hours before arriving at the laboratory (Koch & Stern, 2004; Wolpert et al., 2020). Right before the training, participants ingested 250 ml of water to stimulate gastric activity. The combination of water ingestion and training likely stimulated the observed increase in normogastria (Koch & Stern, 2004; Stern et al., 2004; van Dyck et al., 2020). After the training, participants performed the two-step water load test (van Dyck et al., 2016) which consists of ingesting water until fullness. Participants’ normogastria significantly decreased afterward, likely due to the large volume of water consumed, which may have caused slight nausea and discomfort (Koch & Stern, 2004). Bradygastria showed an inverse pattern to normogastria, with levels significantly decreasing during training, reaching their lowest point during training, and increasing again post-training. This aligns with previous findings on bradygastria reductions following water ingestion (Diamanti et al., 2003). For tachygastria, the picture is more complex, which will be discussed below.

Regarding group differences, normogastria levels during training were highest in the 2D group and lowest in the VR group, although the differences between groups did not reach significance. For bradygastria, the active groups (VR and 2D) exhibited lower levels of bradygastria than the CG during training, although these group differences did not reach significance either. Although going in the expected direction, these results could not confirm our hypothesis, according to which participants would have the highest normogastria values in the VR group, followed by the 2D group and finally the CG (and an inverse pattern for bradygastria).

For tachygastria, we found a significant decrease from session 1 to session 4, indicating that levels of tachygastria decreased across training sessions. During training, we found a trend for the 2D group, which showed lower levels of tachygastria compared to the VR group, with a medium effect size that was marginally significant. This suggests that the VR condition may have had a stimulating effect, counteracting the relaxation effect of the intervention and leading to higher sympathetic activation and corresponding elevated tachygastria levels (Ruhland et al., 2008). Conversely, participants in the 2D group may have been able to focus on the task without being distracted by VR arousal, resulting in higher relaxation and thus parasympathetic activation (McCorry, 2007) reflected by a decrease in tachygastria (Mazur et al., 2012). As previously reported by Tiemann et al. (2025), which is beyond the scope of the present study, initial results may cautiously support this interpretation, with reduced high frequency normalized units (HFnu) values in the VR compared to the CG and, marginally, compared to the 2D group. The possibility that elevated tachygastria in the VR group was driven by nausea was also considered; however, post-training nausea levels did not differ significantly between groups. Nevertheless, further research is needed to clarify the physiological mechanisms underlying gastric biofeedback.

Our results are difficult to compare to the gastric biofeedback paradigm of Stern et al. (2004) as they did not report sufficient details on bradygastria and tachygastria levels. Nevertheless, they observed a significant increase in normogastria during training in their experimental group compared to the CG, which featured a 2D visualization of a stereotypical 3 cpm wave. While we observed a similar pattern for the 2D group for normogastria, and while bradygastria was lowest in the active groups (VR and 2D), these effects did not reach significance. Tachygastria was lowest in the 2D group, which was marginally significant with a medium effect size compared to the VR group. Although not strongly pronounced, it is still possible that the VR group experienced some degree of VR-related dizziness and nausea, which may have increased tachygastria. Stern and colleagues (2004) reported that there were no differences in tachygastria levels following gastric biofeedback training. It is possible that our training acted more on gastric dysrhythmias, while the training by Stern et al. (2004) had an effect on normogastria. It should also be noted that in the study by Stern et al. (2004), the mean percentage of normogastria during training was substantially lower (between 20 and 35%) compared to the present study (approximately 60–70%). This discrepancy may be attributed, at least in part, to differences in calculation methods. Specifically, we did not adopt the same frequency range for defining normogastria as Stern et al. (2.5–3.75 cpm), but instead followed Wolpert et al. (2020), using a slightly broader range of 2–4 cpm. These methodological differences limit the direct comparability of findings across studies. Another possibility may be that individuals with lower levels of normogastria are capable to increase their levels of normogastria more than individuals who already have high levels (ceiling effect, law of initial values). Notwithstanding these considerations, it seems possible that individuals are capable to reduce their levels of dysrhythmias through gastric biofeedback training (2D group). Regarding the finding that the VR group was not superior to the 2D group, though contrary to our hypothesis, our results are consistent with a recent study on guided breathing in a sample of *N* = 39 healthy young sports students, which did not find additional beneficial effects on physiological changes in VR compared to non-VR biofeedback (Pratviel et al., 2024).

In conclusion, our findings indicate that gastric biofeedback training may help reduce gastric dysrhythmias in healthy young adults, as evidenced by a significant decrease in tachygastria across sessions. The lowest tachygastria levels were observed in the 2D group (medium effect size, marginally significant), suggesting that this condition may be particularly effective, without additional benefit of the VR condition. Additionally, both normogastria and bradygastria followed the expected trajectory from pre-training to post-training, supporting the validity of our EGG measurements. Importantly, gastric biofeedback based on EGG (independent of the mode of delivery) proved to be technically feasible and a low incidence of VR-related nausea reported.

For self-reported experiences, ratings were overall positive, with participants increasingly finding the *visualization of their stomach activity and environmental cues helpful* throughout the sessions. Participants also reported improved *management of physical discomfort* over time across all groups. This may be explained by the fact that all participants were instructed to focus on their gastric sensations, regardless of whether they received feedback. In this sample of healthy young adults, this kind of focused attention alone may have led to enhanced body perception and emotion regulation. This is consistent with recent findings suggesting that attention to gastrointestinal activity can improve body awareness and emotional functioning (Davey et al., 2023). In our case, this may plausibly have supported improved coping with uncomfortable physical sensations.

*General liking*, *concentration*, and *alertness* declined over the sessions, likely due to the long testing sessions (2.5 h for sessions 1 and 4, and 1.5 h for sessions 2 and 3). This decline may be explained by the novelty effect, where users have a preference for novel experiences (Bianchi, 1998; Koch et al., 2018). Greater *spatial presence* was reported in the VR group, which is in line with previous findings regarding spatial presence differences between VR and 2D (Kuhne et al., 2023). However, the 2D and the VR groups were comparable for *involvement* and *realness*. Both VR and 2D training formats demonstrated usefulness in increasing *concentration* and *reducing perceived time*. Overall, the VR did not show significant advantages over 2D regarding training outcomes or self-reported experience – indicating that a 2D version of gastric biofeedback may be a cost-effective and accessible option for broad application.

### Strengths and Limitations

The current study is the first implementation and evaluation of a gastric biofeedback training in VR, demonstrating the technical feasibility and promising effects of this novel method. Participant feedback was generally positive, indicating acceptability and engagement, especially in the first sessions. The study employed a randomized controlled trial design, ensuring comparable groups and reducing bias (Stolberg et al., 2004). A key strength was the inclusion of a highly controlled comparison group, together with a 2D condition that provided an identical visual experience for the VR group, which was missing in past VR biofeedback studies (such as Rockstroh et al., 2019). Nevertheless, there are also important limitations. As already noted, the VR condition may have induced arousal or slight nausea, potentially counteracting the intended relaxation effect of gastric biofeedback. In the future, this could be addressed by allowing more time for participants to habituate to the virtual environment. Moreover, the study lacked an accuracy test to confirm whether participants were truly able to link the feedback to their interoceptive sensations. While the strict control condition in this study enhances internal validity, it is possible that participants actively engaging in imagining and focusing on their stomach activity may have resulted in them undergoing some degree of unintentional training (Davey et al., 2023). Other VR based biofeedback studies used less stringent control conditions, such as inactive control conditions, non-contingent visual input, or plain 2D feedback, making their beneficial effects difficult to compare to stricter control conditions, such as the one in our study (Kerr et al., 2023; Rockstroh et al., 2019). These setups often do not control for participants’ active engagement or expectations, making it difficult to disentangle the specific effects of VR biofeedback. In the present study, however, the control group received a similar degree of structure and instruction but without actual biofeedback, allowing for a more rigorous comparison. This stricter control might explain why differences between groups were smaller than those reported in studies with less controlled baselines.

Our healthy participants may have experienced monotony over time due to repeated exposure to the same visualization, particularly during the relatively lengthy sessions. This could have impacted engagement, as also reflected in the self-report questionnaires. Another possibility is that the observed decline in enjoyment, concentration, and alertness over time among healthy participants reflects the absence of clinical need. In clinical populations, such decreases may be attenuated due to greater symptom burden and treatment motivation. This hypothesis warrants examination in future studies. Another possible adaptation may be to implement shorter but more frequent training sessions, aligned with participants’ individual needs and contexts, may be more feasible and effective in real-world applications. Increasing variety within the environment, offering alternative biofeedback visualizations, or enabling home-based training, particularly if 2D delivery proves sufficient, could further enhance usability and adherence. More sessions may also have supported deeper engagement and learning effects. As a laboratory-based study, our design is subject to the general limitations of such settings (Mitchell, 2012). A larger and more diverse sample would be beneficial in future studies, as the current sample may have been underpowered; observed effect sizes were smaller than the expected medium effects.

Regarding the physiological basis for the present gastric biofeedback training, it is important to acknowledge that surface recordings inherently capture only the electrical signals that reach the skin. While studies comparing simultaneous invasive and non-invasive recordings have shown that surface EGG reliably reflects gastric myoelectrical activity (Hamilton et al., 1986; Lin et al., 2000; Mintchev et al., 1993), there remains some uncertainty about the stomach-specificity of these signals (Wolpert et al., 2020). In particular, it cannot be entirely ruled out that activity from other gastrointestinal organs, especially the colon, may contribute to the recorded signal, as overlapping frequencies between 2 and 12 cpm have been reported (for an overview of the literature, see Wolpert et al., 2020). However, the characteristic 3 cpm rhythm appears to be significantly diminished following gastrectomy but not after removal of the colon, suggesting a predominantly gastric origin (for an overview of the literature, see Wolpert et al., 2020). At the same time, the precise contractile meaning of surface-recorded EGG remains debated, as slow-wave activity does not always translate directly into mechanical contractions and recordings can be affected by movement-related artefacts (Mintchev et al., 1993; Rhee et al., 2011; Sanders et al., 2016; Xing et al., 2006). In this study, participants were instructed to remain still during recordings to minimize such artefacts. The method used in the present study, based on filtering and Fast-Fourier analysis, is well suited for providing real-time feedback on whether gastric activity falls within the normal frequency range, but it is less effective at detecting short-term irregularities in the rhythm (Wolpert et al., 2020). Newer approaches, such as wave-by-wave cycle detection (Wolpert et al., 2020), can capture such irregularities more directly and may offer a useful complement for future gastric biofeedback research. Although invasive methods provide the most accurate measurement results for capturing gastric activity, non-invasive approaches offer important advantages. These include lower cost, greater participant comfort, and the potential for portable or scalable applications beyond medical environments. Future research should aim to validate gastric biofeedback using both methods to refine its physiological specificity.

### Future Research Directions and Clinical Implications

First, reducing participants’ testing burden may be crucial in future research employing the gastric biofeedback paradigm to enhance its positive effects. Future studies could benefit from shorter, fewer and less demanding sessions. Most importantly, future studies should focus on testing the intervention in a clinical population with more pronounced gastric motility issues, as the current sample may have exhibited a ceiling effect, limiting the potential for measurable improvements. Especially in eating disorders, the gastric rhythm seems to be altered (Ogawa et al., 2004; van Dyck et al., 2020). Applying gastric biofeedback in the treatment of patients with eating disorders may be a valuable application of the current paradigm. Patients with depression (Ruhland et al., 2008), diabetes (Mathur et al., 2001), schizophrenia (Peupelmann et al., 2009) or other conditions that are accompanied by disturbances of the gastric rhythm could potentially also benefit from the intervention.

### Conclusions

This study demonstrates that a novel gastric biofeedback paradigm is technically feasible and well accepted by participants. Expected changes in gastric activity during the sessions could be clearly generated. We observed group effects for time perception and concentration, with time passing faster in the 2D group compared to controls, and higher concentration in the biofeedback groups (2D and VR). All participants reported improvements in dealing with physical discomfort over time. Additionally, the training appears potentially promising in reducing dysrhythmic gastric activity (tachygastria) over time. We found a trend according to which the 2D group (and not the VR group) showed the greatest improvements, indicating that this format might be promising. It also suggests that a higher sense of presence in the scene might not be required for gastric biofeedback success in this sample. Yet, future studies will need to explore this further. These results offer new insights into the feasibility, efficacy, and experience of gastric biofeedback training, a perspective not yet extensively evaluated. Future research should focus on testing clinical populations to further confirm its efficacy.

## Supplementary Information

Below is the link to the electronic supplementary material.

Supplementary Material 1 (DOCX 80kB)

## Data Availability

The dataset is available in the OSF repository. https://osf.io/en46g/files/osfstorage/6887a1c38d7a95e7fea23f96.
