# Supplementary material for: Gastric Biofeedback in Virtual Reality: Feasibility, Efficacy and Self-Reported Experience
Source: Appl Psychophysiol Biofeedback. 2025 Oct 23;51(2):405–21. doi: 10.1007/s10484-025-09741-x (PMC13179254; doi:10.1007/s10484-025-09741-x)
Supplement: Supplementary file 1 — Supplementary Material 1 (DOCX 80kB) [file 10484_2025_9741_MOESM1_ESM.docx]

**Supplement S1**

***Instructions for the VR Biofeedback condition and 2D condition (German)***

*«* Das Wahrnehmen von körpereigenen Signalen hängt mit gesundem Essverhalten, gesundem Körpergewicht und Gesundheit im Allgemeinen zusammen. Haben Sie daher im Alltag ruhig Vertrauen, dass Ihre Körpersignale (wie z.B. Magensignale) vertrauenswürdige und nützliche Informationen liefern können.

Wir können nun zur nächsten Aufgabe übergehen. In dieser Aufgabe geht es darum zu untersuchen, in welchem Ausmass Personen in der Lage sind, ihre Magenaktivität durch Entspannung und geistige Anstrengung zu beeinflussen.

Normale Magenaktivität besteht aus drei Kontraktionen die Minute und das Ziel ist es, dass Sie versuchen, Ihren Anteil an normaler Magenaktivität zu erhöhen. Sie werden nun Ihre eigene Magenaktivität in einer virtuellen Naturumgebung über das *Virtual-Reality-Headset* / *den Fernseher* sehen. Es gibt zwei durchsichtige Kugeln, die sich rhythmisch mit Wasser füllen und wieder entleeren: die Linke zeigt eine Aktivität von dreimal die Minute an, die als "Schrittmacher" fungiert, und die Rechte zeigt Ihre eigene Magenaktivität an.

Ihr Ziel ist es, Ihre eigene Aktivität an den Schrittmacher anzupassen. Dies können Sie durch Entspannung und regelmässiges, ruhiges Atmen schaffen. Die Umgebung (Wolken, Wasserfarbe und Windgeräusche) ist nach 6-7 min proportional zu wie sehr sie sich entspannen und ihre Magenaktivität sich dem Beispiel anpasst (je schönes das Wetter, desto mehr passen sich ihre Aktivität an das Beispiel an). Lassen Sie sich bei der Aufgabe nicht stressen, wenn sich die Kugeln nicht perfekt angleichen (z.B. kann es sein, dass das Wasser in der Kugel nicht ganz hoch oder ganz runter geht). Es ist normal, dass sie nicht ganz gleich sind und perfekt übereinstimmen, wir sind ja auch nur Menschen. Es geht nur darum, dass sie sich annähern und ähnlicher werden. Denken Sie auch wieder dran, sich so wenig wie möglich zu bewegen, da wir die physiologischen Daten sonst nicht benutzen können.»

«Ist alles klar oder gibt es noch Fragen?» «Können Sie mir bitte kurz zusammenfassen, was Sie verstanden haben, was sie tun sollen?» «Können Sie mir kurz sagen, was die 2 Kugeln Abbilden?» «Können Sie mir kurz zusammenfassen, was Sie machen sollen? Und was das Wetter zeigt nach 6-7 min?»

***Instructions for the control condition (German)***

«Das Wahrnehmen von körpereigenen Signalen hängt mit gesundem Essverhalten, gesundem Körpergewicht und Gesundheit im Allgemeinen zusammen. Haben Sie daher im Alltag ruhig Vertrauen, dass Ihre Körpersignale (wie z.B. Magensignale) vertrauenswürdige und nützliche Informationen liefern können.

Wir können nun zur nächsten Aufgabe übergehen. In dieser Aufgabe geht es darum zu untersuchen, in welchem Ausmass Personen in der Lage sind, ihre Magenaktivität durch Entspannung und geistige Anstrengung zu beeinflussen.

Normale Magenaktivität besteht aus drei Kontraktionen die Minute und das Ziel ist es, dass Sie versuchen, Ihren Anteil an normaler Magenaktivität zu erhöhen. Dies können Sie erreichen, indem Sie sich entspannen, regelmässig und ruhig atmen, und an schöne Dinge denken, wie beispielsweise an einem schönen Tag am Strand zu liegen. Stellen Sie sich ebenfalls vor, dass Ihr Magen sich 3-mal die Minute zusammenzieht, wie ein Luftballon der sich dreimal pro Minute aufbläst und wieder entleert, oder wie ein mit Wasser gefüllter Ball, der sich dreimal pro Minute füllt und entleert. Bitte versuchen Sie auch hier, sich möglichst wenig zu bewegen, die Kontraktionen der Bauchmuskeln zu minimieren und nicht zu sprechen.»

«Ist alles klar oder gibt es noch Fragen?» «Können Sie mir bitte kurz zusammenfassen, was Sie verstanden haben, was sie tun sollen?»

| **Supplement S2**  **Table 2**  *Descriptive statistics of gastric data and self-reported experience measures* | | | | | | | | | | | | | | | | | | | | |
| --- | --- | --- | --- | --- | --- | --- | --- | --- | --- | --- | --- | --- | --- | --- | --- | --- | --- | --- | --- | --- |
|  | **VR** | | | | | **2D** | | | | | **CG** | | | | | **Total** | | | | |
|  | *n* | *M* | *SD* | Min | Max | *n* | *M* | *SD* | Min | Max | *n* | *M* | *SD* | Min | Max | *n* | *M* | *SD* | Min | Max |
| Bradygastria pre-training | 114 | 22.49 | 13.42 | 1.59 | 61.76 | 118 | 26.40 | 14.98 | 2.76 | 65.48 | 121 | 25.39 | 13.64 | 2.08 | 63.55 | 353 | 24.79 | 14.09 | 1.59 | 65.48 |
| Normogastria pre-training | 114 | 56.09 | 17.07 | 18.72 | 92.58 | 118 | 53.21 | 14.98 | 23.77 | 84.14 | 121 | 52.18 | 15.12 | 18.18 | 94.75 | 353 | 53.79 | 15.77 | 18.18 | 94.75 |
| Tachygastria pre-training | 114 | 21.42 | 11.25 | 5.39 | 62.70 | 118 | 20.38 | 10.49 | 4.55 | 53.65 | 121 | 22.44 | 12.06 | 3.17 | 77.96 | 353 | 21.42 | 11.29 | 3.17 | 77.96 |
| Bradygastria training | 119 | 9.25 | 6.80 | 0.98 | 35.84 | 125 | 10.40 | 5.84 | 1.74 | 32.99 | 104 | 11.40 | 6.52 | 3.04 | 33.46 | 358 | 10.31 | 6.42 | 0.98 | 35.84 |
| Normogastria training | 119 | 64.83 | 18.99 | 7.32 | 92.96 | 125 | 69.97 | 13.76 | 20.22 | 94.04 | 104 | 68.55 | 14.14 | 28.83 | 91.62 | 358 | 67.79 | 15.95 | 7.32 | 94.04 |
| Tachygastria training | 119 | 25.92 | 19.33 | 5.06 | 91.40 | 125 | 19.63 | 12.63 | 3.50 | 72.84 | 104 | 20.05 | 13.06 | 3.96 | 64.43 | 358 | 21.91 | 15.59 | 3.50 | 91.40 |
| Bradygastria post-training | 116 | 19.00 | 10.60 | 2.34 | 53.11 | 121 | 20.56 | 11.35 | 2.71 | 56.77 | 122 | 19.12 | 11.60 | 1.77 | 56.96 | 359 | 19.57 | 11.19 | 1.77 | 56.96 |
| Normogastria post-training | 116 | 60.85 | 15.34 | 26.61 | 93.67 | 121 | 58.41 | 14.03 | 24.58 | 90.93 | 122 | 61.11 | 15.90 | 29.15 | 94.33 | 359 | 60.12 | 15.12 | 24.58 | 94.33 |
| Tachygastria post-training | 116 | 20.15 | 9.73 | 2.77 | 48.71 | 121 | 21.03 | 9.76 | 4.96 | 46.27 | 122 | 19.77 | 11.88 | 1.96 | 66.91 | 359 | 20.32 | 10.51 | 1.96 | 66.91 |
| Scene relaxing | 119 | 7.88 | 1.64 | 2.00 | 10 | 127 | 7.84 | 1.66 | 3.00 | 10 | / | / | / | / | / | 246 | 7.86 | 1.65 | 2.00 | 10 |
| Sphere intuitive visualization | 119 | 7.10 | 1.93 | 2.00 | 10 | 127 | 6.54 | 2.09 | 1.00 | 10 | / | / | / | / | / | 246 | 6.81 | 2.03 | 1.00 | 10 |
| Environment helpful | 119 | 6.07 | 2.55 | 0.00 | 10 | 127 | 5.82 | 2.60 | 1.00 | 10 | / | / | / | / | / | 246 | 5.94 | 2.57 | 0.00 | 10 |
| *Note.* **2D** = 2D Group (television). **VR** = Virtual Reality Group. **CG** = Control Group. ***M*** = Mean. ***SD*** = Standard Deviation. | | | | | | | | | | | | | | | | | | | | |

| **Supplement S2 (continued)**  **Table 2**  *Descriptive statistics of gastric data and self-reported experience measures* | | | | | | | | | | | | | | | | | | | | |
| --- | --- | --- | --- | --- | --- | --- | --- | --- | --- | --- | --- | --- | --- | --- | --- | --- | --- | --- | --- | --- |
|  | **VR** | | | | | **2D** | | | | | **CG** | | | | | **Total** | | | | |
|  | *n* | *M* | *SD* | Min | Max | *n* | *M* | *SD* | Min | Max | *n* | *M* | *SD* | Min | Max | *n* | *M* | *SD* | *Min* | *Max* |
| Liking | 119 | 7.10 | 1.94 | 1.00 | 10 | 128 | 7.21 | 1.91 | 1.00 | 10 | 128 | 6.59 | 2.64 | 0.00 | 10 | 375 | 6.96 | 2.20 | 0.00 | 10 |
| Intention to use | 119 | 4.55 | 2.62 | 0.00 | 10 | 128 | 4.38 | 2.88 | 0.00 | 10 | 128 | 4.67 | 2.77 | 0.00 | 10 | 375 | 4.53 | 2.76 | 0.00 | 10 |
| Recommendation to use | 119 | 5.27 | 2.38 | 0.00 | 10 | 128 | 5.80 | 2.16 | 1.00 | 10 | 128 | 5.67 | 2.67 | 0.00 | 10 | 375 | 5.59 | 4.42 | 0.00 | 10 |
| Time perception | 119 | 6.81 | 2.18 | 1.00 | 10 | 128 | 6.88 | 2.12 | 1.00 | 10 | 128 | 5.84 | 2.67 | 0.00 | 10 | 375 | 6.50 | 2.38 | 0.00 | 10 |
| Concentration | 119 | 8.03 | 1.91 | 1.00 | 10 | 128 | 8.00 | 1.62 | 3.00 | 10 | 128 | 7.23 | 2.01 | 1.00 | 10 | 375 | 7.75 | 1.89 | 1.00 | 10 |
| Distraction | 119 | 2.50 | 2.41 | 0.00 | 9.00 | 128 | 3.07 | 2.49 | 0.00 | 10 | 128 | 3.62 | 2.61 | 0.00 | 10 | 375 | 3.08 | 2.54 | 0.00 | 10 |
| MBDF good mood | 119 | 2.62 | 0.28 | 2.00 | 3.50 | 128 | 2.63 | 0.35 | 1.75 | 3.50 | 128 | 2.64 | 0.29 | 1.50 | 3.25 | 375 | 2.63 | 0.31 | 1.50 | 3.50 |
| MBDF alert tired | 119 | 2.80 | 0.59 | 1.80 | 4.67 | 128 | 2.94 | 0.66 | 1.60 | 4.67 | 128 | 2.83 | 0.54 | 1.80 | 4.33 | 375 | 2.86 | 0.60 | 1.60 | 4.67 |
| MBDF rest unrest | 119 | 2.78 | 0.37 | 2.00 | 3.75 | 128 | 2.83 | 0.38 | 1.75 | 4.00 | 128 | 2.75 | 0.38 | 1.50 | 3.50 | 375 | 2.79 | 0.38 | 1.50 | 4.00 |
| IPQ realism | 107 | 2.30 | 0.90 | 0.33 | 4.67 | 103 | 2.26 | 1.08 | 0.33 | 5.00 | / | / | / | / | / | 210 | 2.28 | 0.99 | 0.33 | 5.00 |
| IPQ spatial presence | 107 | 3.2 | 1.29 | 0.40 | 6.00 | 103 | 2.25 | 1.20 | 0.40 | 6.00 | / | / | / | / | / | 210 | 2.73 | 1.33 | 0.40 | 6.00 |
| IPQ involvement | 107 | 3.17 | 1.28 | 0.50 | 6.00 | 103 | 2.86 | 1.24 | 0.50 | 5.25 | / | / | / | / | / | 210 | 3.02 | 1.27 | 0.50 | 6.00 |
| VRSQ mean | 119 | 1.64 | 0.44 | 1.00 | 2.78 | 128 | 1.60 | 0.42 | 1.00 | 2.89 | 94 | 1.62 | 0.42 | 1.00 | 3.00 | 341 | 1.62 | 0.43 | 1.00 | 3.00 |
| Nausea | 118 | 1.30 | 0.71 | 1 | 5 | 128 | 1.51 | 1.04 | 1 | 5 | 128 | 1.46 | 1.08 | 1 | 6 | 374 | 1.43 | 0.97 | 1 | 6 |
| UAQ mean | 119 | 3.05 | 0.64 | 1.43 | 4.14 | 128 | 3.01 | 0.74 | 1.57 | 4.71 | 128 | 2.97 | 0.64 | 1.71 | 4.43 | 375 | 3.01 | 0.67 | 1.43 | 4.71 |
| *Note.* **2D** = 2D Group (television). **VR** = Virtual Reality Group. **CG** = Control Group. ***M*** = Mean. ***SD*** = Standard Deviation. | | | | | | | | | | | | | | | | | | | | |

**Supplement S3**

**Table 3**

*Significant Post Hoc Comparisons for Timepoint × Group Interaction of Hypothesis 1: Normogastria Timepoint and Group Analysis*

| *Contrast* | *p* | *d* | *95% CI for d* |
| --- | --- | --- | --- |
| Baseline VR < Training VR | < .001 | -0.63 | [-0.89, -0.37] |
| Baseline VR < Training 2D | < .001 | -1.06 | [-1.42, -0.70] |
| Baseline VR < Training CG | .0003 | -0.86 | [-1.23, -0.49] |
| Training VR > Baseline 2D | .0005 | 0.80 | [0.44, 1.16] |
| Training VR > Baseline CG | < .001 | 0.91 | [0.55, 1.27] |
| Post VR < Training 2D | .0035 | -0.71 | [-1.07, -0.35] |
| Post VR > Baseline CG | .0199 | 0.63 | [0.27, 0.99] |
| Baseline 2D < Training 2D | < .001 | -1.23 | [-1.48, -0.97] |
| Baseline 2D < Training CG | < .001 | -1.03 | [-1.40, -0.66] |
| Training 2D > Post 2D | < .001 | 0.85 | [0.60, 1.11] |
| Training 2D > Baseline CG | < .001 | 1.34 | [0.99, 1.70] |
| Training 2D > Post CG | .0048 | 0.69 | [0.34, 1.05] |
| Post 2D > Training CG | .0157 | -0.65 | [-1.02, -0.29] |
| Baseline CG < Training CG | < .001 | -1.14 | [-1.41, -0.87] |
| Baseline CG < Post CG | < .001 | -0.65 | [-0.90, -0.40] |
| Training CG > Post CG | .0091 | 0.49 | [0.22, 0.76] |

| **Supplement S4**  **Table 4**  *Mixed-Model ANOVA results for self-reported variables* | | | | | | | | | | |
| --- | --- | --- | --- | --- | --- | --- | --- | --- | --- | --- |
|  | **Session / Timepoint** | | | | **Group** | | | **Session / Timepoint  × Group** | | |
|  | df | F | | *p* | df | F | *p* | df | F | *p* |
| Scene relaxing | 3, 178.45 | | 0.59 | .622 | 1, 67.40 | 0.01 | .931 | 3, 178.68 | 0.62 | .600 |
| Sphere intuitive visualization | 3, 178.53 | | 1.89 | .132 | 1, 67.73 | 1.85 | .179 | 3, 178.75 | 1.80 | .149 |
| Environment helpful | 3, 178.01 | | 4.92 | .003 | 1, 72.80 | 0.08 | .784 | 3, 178.05 | 0.27 | .848 |
| Liking | 3, 272.06 | | 3.71 | .012 | 2, 101.52 | 1.10 | .338 | 6, 272.07 | 0.95 | .458 |
| Intention to use | 3, 272.12 | | 0.36 | .779 | 2, 104.12 | 0.07 | .936 | 6, 272.08 | 0.82 | .554 |
| Recommendation to use | 3, 272.22 | | 0.26 | .854 | 2, 102.64 | 0.69 | .506 | 6, 272.21 | 0.90 | .494 |
| Time perception | 3, 272.07 | | 2.47 | .062 | 2, 94.93 | 3.59 | .031 | 6, 272.33 | 0.83 | .545 |
| Concentration | 3, 272.13 | | 7.30 | < .001 | 2, 95.98 | 3.21 | .045 | 6, 272.31 | 1.36 | .233 |
| Distraction | 3, 272.23 | | 1.60 | .190 | 2, 96.43 | 2.43 | .093 | 6, 272.40 | 0.67 | .672 |
| MBDF good mood | 3, 272.42 | | 1.06 | .369 | 2, 93.68 | 0.06 | .944 | 6, 272.87 | 0.77 | .597 |
| MBDF alert tired | 3, 272.36 | | 4.28 | .006 | 2, 93.53 | 0.93 | .397 | 6, 272.83 | 0.90 | .496 |
| MBDF rest unrest | 3, 272.53 | | 1.41 | .240 | 2, 93.45 | 0.82 | .445 | 6, 273.06 | 1.28 | .266 |
| IPQ realism | 3, 151.26 | | 0.73 | .533 | 1, 66.33 | 0.002 | .963 | 3, 151.25 | 2.30 | .079 |
| IPQ spatial presence | 3, 150.93 | | 0.87 | .457 | 1, 66.56 | 8.44 | .005 | 3, 150.90 | 1.34 | .263 |
| IPQ involvement | 3, 151.29 | | 1.92 | .129 | 1, 57.90 | 0.74 | .392 | 3, 151.52 | 1.34 | .265 |
| VRSQ mean | 3, 242.12 | | 2.11 | .099 | 2, 86.39 | 0.17 | .843 | 6, 242.12 | 2.57 | .020 |
| Nausea | 3, 271.43 | | 4.85 | .003 | 2, 96.11 | 0.45 | .639 | 6, 271.60 | 1.03 | .407 |
| UAQ1 Physical symptom improvement | 3, 272.11 | | 8.43 | < .001 | 2, 98.59 | 0.65 | .523 | 6, 272.19 | 0.94 | .464 |
| UAQ2 physical well-being improvement | 3, 271.86 | | 1.46 | .226 | 2, 97.05 | 0.79 | .455 | 6, 271.98 | 0.95 | .461 |
| UAQ3 positive impact on mood | 3, 271.93 | | 0.39 | .757 | 2, 98.45 | 0.03 | .966 | 6, 272.00 | 0.98 | .441 |
| UAQ4 positive impact on experience of stress | 3, 271.31 | | 0.85 | .466 | 2, 97.01 | 0.00 | .998 | 6, 271.41 | 0.42 | .864 |
| UAQ5 overall training satisfaction | 3, 272.11 | | 1.57 | .198 | 2, 98.73 | 0.67 | .515 | 6, 272.18 | 0.81 | .565 |
| UAQ6 applying of strategies in everyday life | 3, 271.64 | | 0.54 | .658 | 2, 98.13 | 0.02 | .979 | 6, 271.71 | 0.85 | .530 |
| UAQ7 side effects | 3, 271.81 | | 0.87 | .455 | 2, 94.10 | 0.39 | .679 | 6, 272.12 | 1.64 | .136 |
| *Note.* **BMI** = Body Mass Index. **DASS** = Depression-Anxiety-Stress Scale – 21 (Nilges & Essau,2021). **EDIP** = Eating disorder-specific interoceptive perception questionnaire (Ortmann et al.,2024). **HBCT** = heart beat counting task (Legrand et al.,2022). **HFnu** = high frequency normalized units. **HR** = heart rate. **HRV** = heart rate variability. **MAIA-2** = Multidimensional Assessment of Interoceptive Awareness questionnaire 2 (Mehling et al.,2018b). **RMSSD** = root mean square of successive differences. **WLT-II** = two-step water load test (van Dyck,Vögele,et al.,2016) | | | | | | | | | | |
